# Adhesion and Cohesion

**DOI:** 10.1155/2012/951324

**Published:** 2012-02-21

**Authors:** J. Anthony von Fraunhofer

**Affiliations:** School of Dentistry, University of Maryland, Baltimore, MD 21201, USA

## Abstract

The phenomena of adhesion and cohesion are reviewed and discussed with particular reference to dentistry. This review considers the forces involved in cohesion and adhesion together with the mechanisms of adhesion and the underlying molecular processes involved in bonding of dissimilar materials. The forces involved in surface tension, surface wetting, chemical adhesion, dispersive adhesion, diffusive adhesion, and mechanical adhesion are reviewed in detail and examples relevant to adhesive dentistry and bonding are given. Substrate surface chemistry and its influence on adhesion, together with the properties of adhesive materials, are evaluated. The underlying mechanisms involved in adhesion failure are covered. The relevance of the adhesion zone and its importance with regard to adhesive dentistry and bonding to enamel and dentin is discussed.

## 1. Introduction

**Figure 1:**
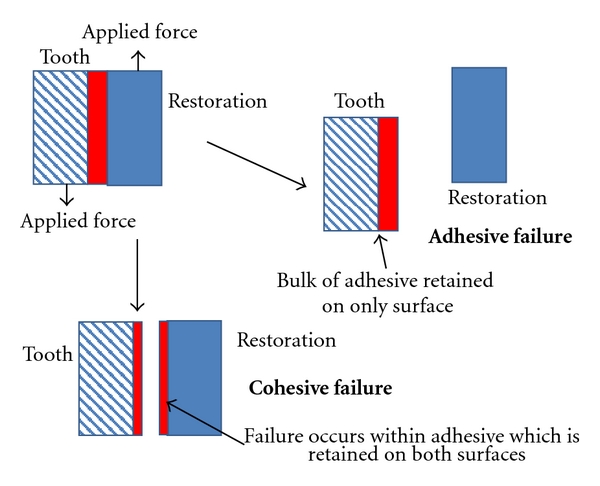
Adhesive and cohesive joint failures.

Every clinician has experienced the failure of a restoration, be it loosening of a crown, loss of an anterior Class V restoration, or leakage of a composite restoration. The procedure is much the same for any such failure, namely, removal of residual adhesive or luting agent and recementation of the restoration. The clinical notes will describe the problem as, commonly, adhesive or cohesive failure based on a simple classification system such as that in Figure 1. The causes of such failures are seldom addressed by most clinicians.

Adhesion and cohesion are terms that are often confused although these subjects are discussed in many standard texts in dental biomaterials science [1–3]. There are also many excellent texts and monographs on adhesion, cohesion, and interfacial reactions [4–6] together with a comprehensive treatment in the on-line encyclopedia, Wikipedia. Since adhesion and cohesion play a very important role in the use of luting agents, an in-depth discussion is appropriate in view of the communications presented in this issue.

The Merriam-Webster dictionary has several definitions of the word “adhesion” but the most apposite here is *the molecular attraction exerted between the surfaces of bodies in contact*. This dictionary likewise has several definitions of the word “cohesion” but the most pertinent here is *the molecular attraction by which the particles of a body are united throughout the mass. *In other words, adhesion is any attraction process between dissimilar molecular species, which have been brought into direct contact such that the adhesive “clings” or binds to the applied surface or substrate. The postsurgical complication of adhesions, involving soft tissues, will not be discussed here.

**Figure 2:**
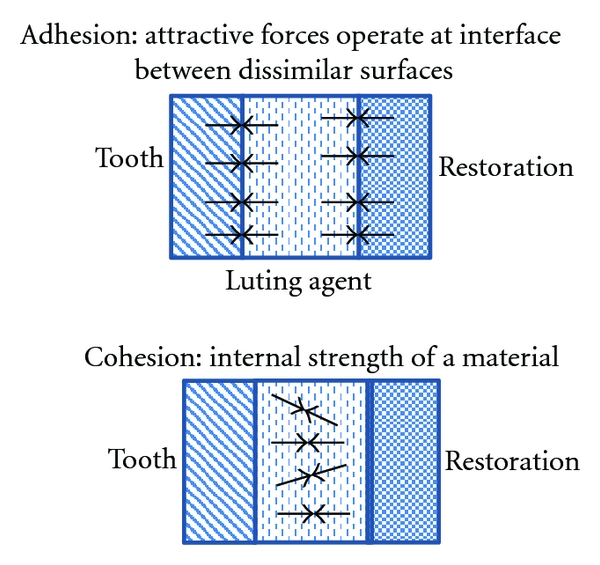
Adhesion and cohesion.

**Figure 3:**
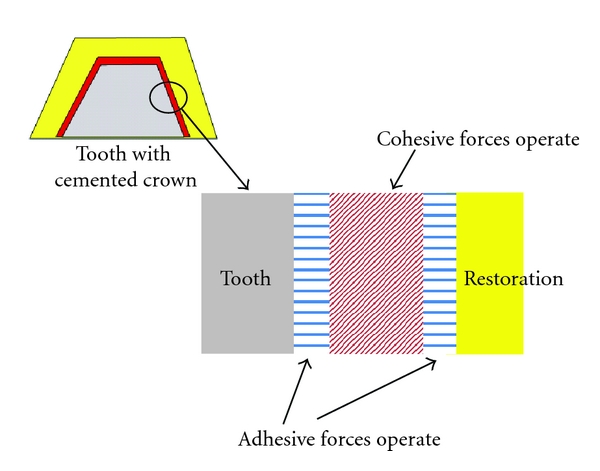
Adhesive and cohesive forces operating within a cemented restoration.

In contrast, cohesion is an attraction process that occurs between similar molecules, primarily as the result of chemical bonds that have formed between the individual components of the adhesive or luting agent. Thus, cohesion may be defined as the internal strength of an adhesive due to various interactions within that adhesive that binds the mass together, whereas adhesion is the bonding of one material to another, namely, an adhesive to a substrate, due to a number of different possible interactions at the adhesive-substrate surface interface. These differences are shown schematically in Figure 2. In dentistry, when a restoration is cemented or bonded to a tooth, adhesive forces bind the luting agent to the restoration on one side and to the tooth on the other side with cohesive forces operating within the luting agent itself, Figure 3.

The characteristics of chewing and bubble gums clearly indicate the difference between cohesion and adhesion. Gum holds together during mastication because of good cohesion and, in the case of bubble gum, enables the gum to be blown into a bubble. These materials, however, exhibit poor adhesion in that they do not readily stick to the teeth, oral tissues, or other surfaces, unless mechanical effects intervene. If, for example, gum while being chewed can lodge into undercuts or between teeth, it can get locked in and may be torn away from the bulk of the gum, that is, mechanical interlocking of the gum within the interproximal area is greater than the cohesive strength of the gum. Likewise, chewed gum does not stick well to smooth surfaces such as glass or polished metal because of its poor adhesion. However, if the masticated and softened gum is pressed onto a rough surface, the gum will distort and flow into gaps, rugosity and voids in the surface such that it “sticks,” often very tightly, to that surface, as most of us know when we try to scrape discarded gum off the soles of our shoes.

Likewise, zinc phosphate cement has good cohesive strength but exhibits poor adhesion to smooth surfaces. In particular, it does not bond, chemically to surfaces and its bonding or adhesion, that is, its application as a luting agent, is possible only through mechanical interlocking at the interface with the restoration and that with the tooth. Zinc phosphate cement, however, does possess good cohesive strength, even in thin films, so that when used as a luting agent for restorations subject to high masticatory stresses, it can support elastic deformation [7].

In every situation involving an adhesive and a substrate, the combination of adhesion and cohesion determines the overall bonding effectiveness. The adhesive bond will fail if the adhesive separates from the substrate or there is internal breakdown of the adhesive (i.e., cohesive failure), Figure 1.

## 2. Forces in Cohesion

 The cohesive strength of a luting agent or adhesive, regardless of its chemical composition, is determined by a number of molecular forces:

1. the chemical bonds within the adhesive material,
2. chemical bonds due to crosslinking of the polymer(s) within a resin-based material,
3. intermolecular interactions between the adhesive molecules, and
4. mechanical bonds and interactions between the molecules in the adhesive.

These molecular interactions, really intermolecular forces, affect the properties of the uncured (unset) adhesive, typically the consistency, flow properties, and viscosity of the adhesive. When the adhesive sets or “cures” to a solid mass, solidification occurs through bonds formed between the molecules in the adhesive, through formation of new bonds and by strengthening of existing bonds. This overall process typically consists of crosslinking of short chain molecules to form longer chains and/or formation of 3-dimensional networks of molecular chains. The latter is the common mechanism involved in the setting of zinc oxide-based dental cements. It follows from this that the cohesive strength of an adhesive is significantly affected by the curing conditions and, when curing/setting occurs under suboptimal conditions, the adhesive will lack cohesive strength.

Suboptimal conditions during the setting or solidification process is a common concern in restorative dentistry and all luting agents, regardless of composition and characteristics, must be protected against the effects of oral fluids prior to and during the curing process to avoid detrimental effects on the setting reactions. Ingress of saliva and oral fluids into the adhesive during the setting process will adversely affect the curing reactions of both inorganic and organic adhesive materials, commonly reducing strength, bonding efficiency and the degree of cure. Thus, fluid ingress will not only imperil the integrity and efficacy of the adhesive-substrate interactions at both the tooth and restoration interfaces but also decrease the cohesive strength of the adhesive. The latter effect is important because the maximum load a bond can withstand in clinical practice as well as in laboratory strength tests may be dictated primarily by the cohesive strength of the adhesive, that is, under loading, the bond fractures due to cohesive failure of the adhesive rather than failure of the adhesive-substrate bond. In other words, the cohesive strength of the adhesive, and not the adhesion between adhesive and the substrate, may be the limiting factor in bond strength tests and in clinical practice.

## 3. Forces in Adhesion

 Adhesion is the propensity of dissimilar particles and/or surfaces to adhere or bond to one another and can be divided into three basic types, Table 1. Specific adhesion is achieved through molecular interactions between the adhesive and the substrate surface. The intermolecular forces produce specific adhesion although this can really be divided into three different types, namely, chemical adhesion, dispersive adhesion, and diffusive adhesion, to which are added mechanical effects in effective adhesion. However, a distinction must be made between weak intermolecular interactions and strong chemical bonds. Although chemical bonds can form in a few substrate/adhesive combinations, for example, epoxy resin and aluminum, they are generally uncommon in dentistry except for those that occur between carboxylate-based luting agents and the calcium within dental hard tissues. When there are chemical bonds within the adhesive joints, they can account for up to 50% of all interactions although the long-term stability of these bonds is usually dependent on their resistance to moisture.

 In addition to the intermolecular and chemical adhesion forces, micromechanical adhesion also can be involved in the overall adhesion phenomenon. In such cases, the adhesive can effectively cling to a roughened substrate surface and increase overall adhesion, for example, chewing gum attached to the soles of our shoes.

## 4. Mechanisms of Adhesion

 The strength of the adhesion between two materials depends on the interactions between the two materials, and the surface area over which the two materials are in contact. As a result, a number of factors enter into the overall adhesion system.

### 4.1. Contact Angle and Surface Tension

 Materials that wet against each other tend to have a larger contact area than those that do not, however, wetting depends on the relative surface energies of the adhesive and substrate materials. Low surface energy materials such as poly(tetrafluoroethylene) or PTFE and silicone materials do not wet and are resistant to adhesive bonding without special surface preparation, hence the use of these polymers to manufacture nonstick cookware and other nonstick surfaces.

**Figure 4:**
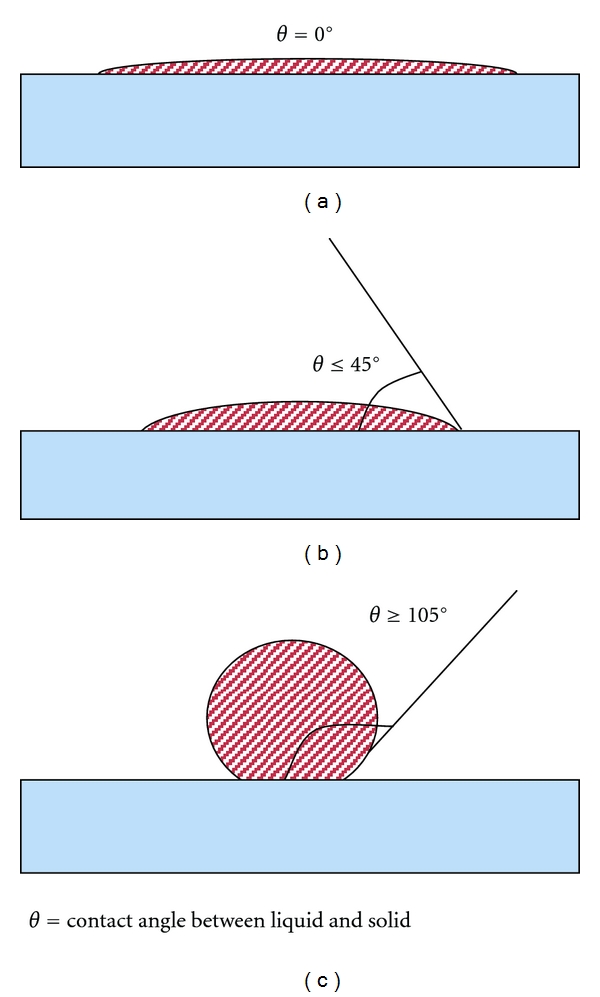
Liquid/surface contact angles for clean, slightly contaminated, and contaminated surfaces.

 Wetting is the ability of a liquid to form an interface with a solid surface and the degree of wetting is evaluated as the contact angle *θ* formed between the liquid and the solid substrate surface. This is determined by both the surface tension of the liquid and the nature and condition of the substrate surface. The smaller the contact angle and the lower the surface tension of the liquid, the greater the degree of wetting, that is, the droplet of liquid will spread across the substrate surface provided the latter is clean and uncontaminated, as shown in Figure 4. A clean surface allows good wetting, that is, the contact angle *θ* is close to 0°, Figure 4(a). There will be a greater contact angle (*θ* is greater than 0° but less than 90°, i.e., 0° < *θ* < 90°) with a slightly contaminated surface, Figure 4(b), and the contact angle between the liquid and a contaminated surface or one with low surface energy will exceed 90°, Figure 4(c). The latter condition is sometimes referred to as dewetting and the liquid will form droplets on the substrate surface.

The contact angle *θ* is a function of both dispersive adhesion (the interaction between the molecules in the adhesive and those of the solid, as discussed later) and the cohesion within the liquid adhesive. If there is strong adhesion to the substrate surface and weak cohesion within the liquid, there is a high degree of wetting, often termed lyophilic conditions. Conversely, a combination of weak adhesion and strong cohesion, referred to as lyophobic conditions, results in high contact angles and poor wetting of the substrate surface, that is, droplets form on the surface rather than a film of fluid.

 A small contact angle indicates more adhesion is present because there is a large contact area between the adhesive and the substrate, resulting in a greater overall substrate surface energy and a high interactive force between the liquid and the substrate.

 These relationships can be put in another way. When the surface is wetted, the contact angle is less than 90° (**θ** < 90°), the substrate has high surface energy and the adhesion forces between substrate and liquid are greater than the cohesive forces within the adhesive (i.e., the surface tension of the liquid, *γ*) and the liquid can spread over the substrate surface. If the surface has low energy (or is contaminated), *θ* > 90° and cohesion within the adhesive can exceed the adhesion between liquid and substrate such that there is poor wetting or dewetting, with the liquid forming droplets on the surface.

**Figure 5:**
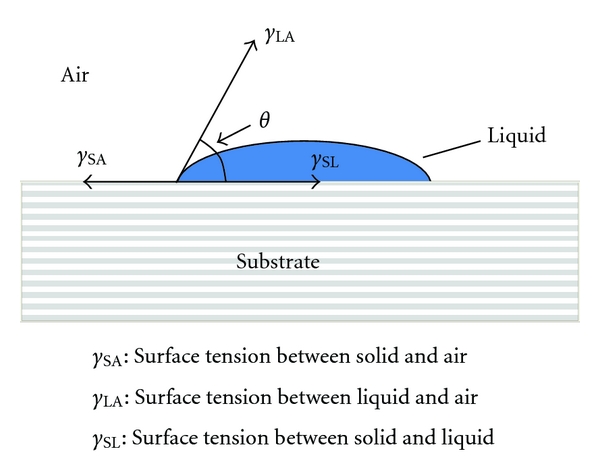
Interfacial tensions for a drop of liquid on a surface.

 Surface scientists express things in a different way and refer to interfacial tension using the terms liquid-air interfacial tension, *γ*_LA_ (i.e., the liquid's surface tension), solid-liquid interfacial tension, *γ*_SL_ (i.e., the surface tension between the solid and the liquid, which approximates to the surface adhesion between liquid and solid) and the solid-air interfacial tension, *γ*_SA_ (i.e., the surface tension between the solid and air, which approximates to the surface energy of the solid), Figure 5.

**Figure 6:**
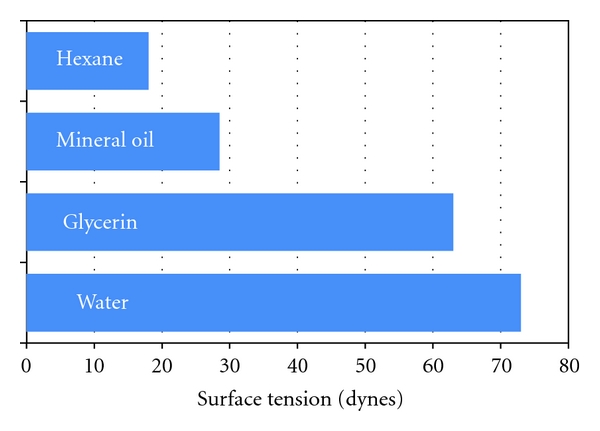
Surface tensions of common liquids.

 Surface tension is commonly expressed as dyne/cm, although it should really be given in the recommended SI units of N/m or J/m².  Figure 6 indicates the relative surfaces tensions of some common liquids.

 For a contact angle of *θ*°, these entities are related by Young's equation,

(1) $$\gamma_{\text{LA}}\cdot \mathrm{Cos}\theta =\gamma_{\text{SA}}-\gamma_{\text{SL}}.$$

If there is complete wetting of the substrate surface, that is, when *θ* = 0 and *Cos*⁡*θ* = 1, Young's equation indicates that *γ*_LA_ = *γ*_SA_ − *γ*_SL_ or *γ*_LA_ ≤ *γ*_SA_. In other words, if the surface tension of the adhesive (*γ*_LA_) is less than the surface energy of the substrate surface (*γ*_SA_), the adhesive will spread over the substrate. For maximum adhesion, the adhesive must completely cover or spread over the substrate, that is, effectively wet it. The contact angle between the adhesive and the substrate is, therefore, a good indicator of adhesive behavior.

**Figure 7:**
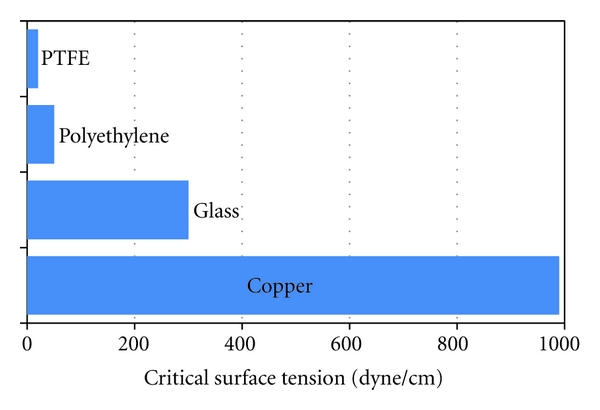
Critical surface tensions of solids (in dyne/cm).

 The value of *γ*_SA_ when *Cos*⁡*θ* = 1 is the critical surface energy (CSE) and equals the value of *γ*_SL_ when the liquid just spreads over the surface. The critical surface tension of several materials is shown in Figure 7. The very large difference in CSE between say glass and PTFE and polyethylene indicates the difficulty of bonding to the two resins.

 Wetting of the surface occurs when the adhesive surface tension (*γ*_SL_) is less than the critical surface energy. This is often expressed as the adhesion quotient which requires the substrate surface energy (*γ*_SA_) to exceed the surface tension of the adhesive liquid (*γ*_SL_) by 10 dyne/cm. If the reverse is true, that is, (*γ*_SL_ ≥ *γ*_SA_), surface wetting is poor, adhesion is reduced and the adhesive tends to pull away from the surface during the curing process.

 The “take home message” here is that the adhesive liquid must wet the substrate surface and such factors as surface contamination, surface conditioning, presence of moisture, and the adhesive used all affect the adhesion between substrate and adhesive. A small contact angle indicates more adhesion is present because there is an interactive force between the liquid and solid phases.

### 4.2. Chemical Adhesion

**Figure 8:**
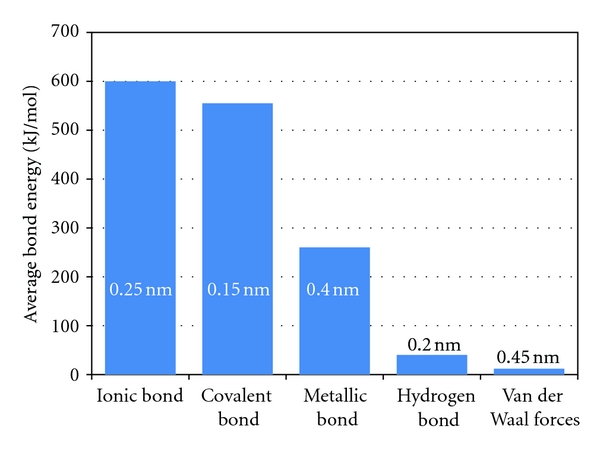
Bond energies and bond lengths of adhesive forces.

 If the adhesive and substrate can form a compound at their interface or union, the ionic or covalent bonds that are formed result in a strong bond between the two materials. A weaker bond is formed when there is hydrogen bonding, that is, a hydrogen atom in one molecule is attracted to an electron-donor atom such as nitrogen or oxygen in another molecule. Thus, when the surface atoms of an adhesive and substrate form ionic, covalent, or hydrogen bonds, chemical adhesion occurs. However, it can be seen that whereas the strengths of these chemical bonds can be high, Figure 8, their lengths are short and therefore for bonding to occur, surfaces with the potential for chemical bonding must be brought very close together and remain in this proximity for the bond to be stable. 

 Although the average lengths of hydrogen bonds are comparable to those of covalent and ionic bonds, they are an order of magnitude weaker. In the case of dental cements, zinc polycarboxylates provide some chemical bonding between the carboxylate molecule of the cement and hydroxyapatite mineral in the tooth, whereas bonding with zinc phosphate cements is wholly mechanical in nature.

### 4.3. Dispersive Adhesion

 In dispersive adhesion or physisorption, the surfaces of two materials are held together by van der Waals forces. The latter are the attractive forces between two molecules, each of which has a region of small positive and negative charge such that the molecules are polar with respect to the average charge density of the molecule; it should be noted that there may be multiple poles (regions of greater positive or negative charge) with larger and/or more complex molecules. If these positive and negative poles are an inherent property of a molecule, they are known as Keesom forces, whereas polarity, that is, a transient effect due to random electron motion within the molecules that cause a temporary concentration of electrons in one region are known as London forces. London dispersion forces, which result from statistical quantum mechanics, are particularly useful in adhesion because they arise without the need for either the adhesive or the substrate surface to have any permanent polarity. Adhesion in surface science commonly refers to dispersive adhesion.

 Although van der Waals bond lengths are longer than those of other molecular forces, see Figure 8, they are still short in absolute terms so that these forces only act over very small distances. About 99% of the work required to break van der Waals bonds is performed once the joined surfaces are separated by more than a nanometer and, as a result, the effectiveness of adhesion due to chemical or dispersive bonding is limited. Once a crack is initiated, it propagates easily along the interface because of the brittle nature of the interfacial bonds and, consequently, greater contact surface areas often provide little difference in the measured adhesion. This topic will be returned to when the adhesion zone is discussed.

### 4.4. Diffusive Adhesion

 Some materials may merge or intermingle at the bonding interface by diffusion, typically when the molecules of both materials are mobile and/or soluble in each other, which typically is the case with polymer chains where one end of a molecule can diffuse into the other material. This form of interaction, known as interdigitation, occurs when a resilient denture liner is processed onto an acrylic resin denture base, or when a fractured denture is repaired with acrylic resin. In such cases, bonding arises from the mutual solubility and interactions between methyl methacrylate (monomer) in the repair (or liner) material and the surface of the poly(methyl methacrylate) or acrylic base with diffusive adhesion (bonding) resulting from sections of polymer chains from the applied material interdigitating with the substrate surface. However, the mobility of the polymers strongly influences their ability to interdigitate to achieve diffusive bonding. Cross-linked polymers are less capable of diffusion and interdigitation because of their restricted mobility whereas non-cross-linked polymers have greater mobility and interdigitate more readily. These differences account for the fact that it is easier to bond a resilient liner to a recently processed acrylic base, or even during processing of the denture base, because the acrylic resin has a greater surface reactivity, that is, there is greater mobility of its surface polymer chains, than when attempting to reline a denture base

 Diffusive adhesion is also the mechanism involved in sintering as, for example, when metal or ceramic powders are compressed and heated so that atoms diffuse from one particle to the next to produce a solid mass. Diffusive bonding occurs when atoms from one surface penetrate into an adjacent surface while still being bound to their surface of origin. This is the mechanism involved in the fusing of porcelain to metal in the fabrication of a PFM crown. Since diffusive adhesion requires interaction of atomic species between two surfaces, the greater the time that the two surfaces can interact, the more diffusion occurs and, accordingly, the stronger the adhesion is between the two surfaces.

### 4.5. Mechanical Adhesion

**Figure 9:**
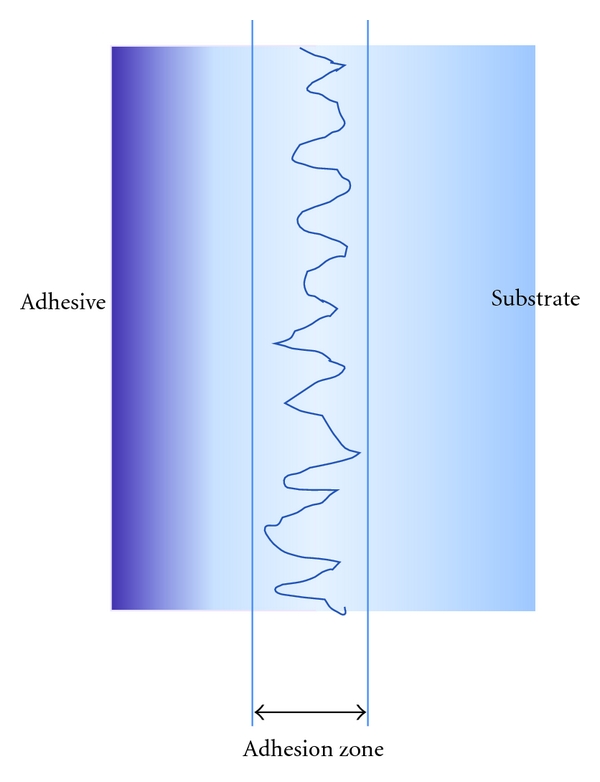
Micromechanical adhesion (schematic).

 When uncured, adhesives are fluid and they can flow over the substrate, filling the voids, rugosity, and pores of the surface and attach or “bond” to that surface by mechanical interlocking. This is often referred to as micromechanical adhesion and is shown schematically in Figure 9.

 Micromechanical adhesion is the primary mechanism for luting of restorations to teeth with dental cements and probably also contributes significantly to bonding achieved with resin-based adhesives as, for example, in fissure sealants and direct bonding of restorative resins. The effectiveness of micromechanical adhesion is determined in large part by the wetting of the substrate by the luting agent in that poor wetting of the substrate by the luting agent will inhibit good apposition of cement and substrate. Further, the luting agent must be able to flow into the surface voids, and so forth, and for this process to occur, the adhesive must have a low viscosity. Water, for example, has a viscosity of 1 centiPoise (cP) and that of alcohol is 1.2 cP. Many other fluids, however, have much higher viscosities, for example, 9.22 cP for eugenol (oil of cloves), 1490 cP for glycerin and ~10^4 ^cP for honey, and the very large difference in the viscosities of honey and water explains why water flows far more readily than honey. It should be noted that the SI units for viscosity are Pa s (Pascal seconds) and are equivalent in magnitude to often quoted cP values.

 Inevitably, micromechanical adhesion of a luting agent to a surface is not simply a matter of wetting (i.e., contact angles) and the rheological or flow properties of the adhesive. Other factors also enter into micromechanical adhesion, notably the electrostatic forces (both attractive and repulsive) that may be operating between the adhesive and the micro-topography of the substrate as well as a property of the applied fluid known as thixotropy. A thixotropic fluid is one that under the action of mechanical forces such as stirring, vibration, and even kneading will temporarily transform to a state that has a lower viscosity and which exhibits better flow than the fluid in its static state. Thixotropic behavior is an important characteristic for endodontic (root canal) sealants which are required to flow into a root canal, often under vibration. Further, thixotropy is often incorporated into industrial and domestic paints by additives such as silicic acid and is probably present in various dental adhesive and cement formulations. Thixotropy, when present in an adhesive, provides certain advantages to the overall adhesion system. In particular, when a thixotropic adhesive is applied to a substrate surface, it will remain in place, even on vertical surfaces. Further, because adhesive flow is determined in part by the mechanical forces imposed on the adhesive, there can be greater control of the adhesive film thickness combined with improved flow into the microtopography of the substrate surface.

## 5. The Adhesion Zone

 It follows from the above that the adhesive bonded to a substrate often has a modified molecular structure at the bonding interface. This interfacial region is known as the adhesion zone (Figure 9) and is characterized by the changes in the adhesive (and sometimes in the substrate) that may arise from the bonding interactions.

The transition zone, the region between the bonding interface and the bulk of the adhesive, is the area over which the chemical, mechanical, and optical properties of the adhesive differ from those of the bulk adhesive. It varies in thickness, from a few nanometers up to a few millimeters, with the thickness depending on the nature of the substrate surface, the chemical composition, and physical characteristics of the adhesive being applied and the curing conditions. Where there are thick transition zones and/or narrow adhesion zones, the behavior of the entire bonding interface may be dependent on the properties of the transition zone because the properties, notably strength, of the adhesive may be impaired because of inadequate cohesion within the adhesive. It is considerations such as these that determine, at least in part, the selection of the optimum luting agent for the various combinations of luting agents and restorations that were discussed by Pameijer in his review of luting agents [8].

## 6. Adhesive Dentistry

Adhesive dentistry, whether it is the cementation (or luting) of a restoration to a prepared tooth or restoration with a composite resin, involves the application and curing of an adhesive at the interface between tooth tissue and the restorative material. Consequently, all of the aspects of adhesion and cohesion discussed above are involved in this process.

 Restoration with a composite material has three principal steps. The first is the creation of microporosity in enamel or dentin by acid etching either through application of an etchant or by the *in situ* action of an etchant/primer/adhesive. The second step is the application of a primer/adhesive which wets and penetrates the created microstructure although because the surface energies of etched enamel and etched dentin differ, different primers are required for the two substrates. Finally, a resin is applied to the primed surface so that when polymerized *in situ*, it micromechanically (i.e., there is mechanical adhesion) interlocks with the substrate microporosity together with a degree of chemical bonding, with some materials exhibiting better chemical adhesion than others.

## 7. Dentin Bonding

 Bonding to dentin presents greater problems than to enamel because it has a high organic content, a non-uniform composition and it is permeated by tubules. Further, after mechanical treatment, a 3–15 *μ*m thick, featureless, and poorly adherent smear layer of organic debris will form. While this smear layer can provide pulpal protection by reducing dentin permeability, it hinders bonding.

 Bonding to dentin involves three stages, namely, conditioning, priming, and bonding, although some commercial bonding systems combine two or more stages into a single step. The conditioning stage involves modifying or removing the smear layer by acidic conditioners, the precise approach being determined by the bonding system used. Priming is the key step in dentin bonding because it promotes interactions between hydrophobic restorative resins and hydrophilic dentin. Primers (dentin bonding agents) are bifunctional molecules, one end being a methacrylate group that bonds to resin and the other a reactive group that reacts with dentin. Thus, primers are coupling agents, that is, they are bifunctional molecules that primarily bond to calcium but may also interact with collagen. The bonding (adhesive) agent is a fluid resin that flows over and wets the primed surface to form an effective bond when cured *in situ*.

 It should be noted that many manufacturers combine many of the conditioning, priming and bonding steps in their systems. If the primer and conditioner are combined as with self-etching primers, the smear layer is incorporated within the primer that directly contacts the dentin and constitutes the adhesive zone. The subsequently applied restorative resin bonds to primed dentin when polymerized. An advantage with self-etching primers is that the dentin is maintained in a moist condition throughout the bonding procedure although enamel etching with such systems is less effective than with phosphoric acid treatment. Alternatively, the primer and adhesive may be combined so that the applied material will infiltrate the collagenous network created by conditioning to form a hybrid (resin-infiltrated reinforced) layer. Subsequently, applied restorative resin, when polymerized, bonds everything together.

 Although high bond strengths (≥20 MPa) to dentin may be achieved, bond failures commonly involve cohesive fracture of the dentin such that these systems are not infallible. They tend to be technique and material sensitive and may require successive treatments for optimal bonding. Further, regardless of high bond strengths, which suggest good adaptation to the dentin, good bonding and the absence of leakage are not synonymous and no system provides consistent leak-free restorations.

## 8. Conclusions

 It follows from the above discussion that the performance of an adhesive in the luting of a restoration to a tooth will be dictated by a multiplicity of factors. Ideally, laboratory bond strength test values and the resistance of luted restorations to clinical loads will be maximized when the propagating crack that causes bond failure has to travel through the adhesion zone rather than the bulk adhesive. In other words, optimal retention is achieved when adhesion rather than the cohesive strength of the adhesive determines the overall strength of the bond [9]. Nevertheless, the mechanical properties of the luting agent often can have a marked impact on the resistance of the luted restoration to applied forces when the thickness of the cement film is markedly greater than the width of the adhesion zone, as noted by *in vivo* determinations of cement film thicknesses beneath restorations [10].

## Figures and Tables

**Table 1:** Basic types of adhesion.

Type	Characteristics
Specific	Molecular attraction between surfaces in contact
Mechanical	Adhesion arising from mechanical interlocking between the adhesive and the substrate surface
Effective	Optimal bonding between adhesive and substrate surface due to combined effects of specific and mechanical adhesion
